# Proteome-wide mendelian randomization identifies causal plasma proteins in interstitial lung disease

**DOI:** 10.1038/s41598-025-85338-y

**Published:** 2025-01-17

**Authors:** Kunrong Yu, Wanying Li, Wenjie Long, Yijia Li, Yanting Li, Huili Liao, Jianhong Liu

**Affiliations:** 1 Guangzhou University of Chinese Medicine, Guangzhou, 510000 China; 2 The First Affiliated Hospital of Guangzhou University of Chinese Medicine, Guangzhou, 510000 China

**Keywords:** Interstitial lung disease, Subtype, Protein, Mendelian randomization, Biomarker, Drug target, Biomarkers, Medical genetics

## Abstract

Interstitial lung disease (ILD) has shown limited treatment advancements, with minimal exploration of circulating protein biomarkers causally linked to ILD and its subtypes beyond idiopathic pulmonary fibrosis (IPF). In this study, we aimed to identify potential drug targets and circulating protein biomarkers for ILD and its subtypes. We utilized the most recent large-scale plasma protein quantitative trait loci (pQTL) data detected from the antibody-based method and ILD and its subtypes’ GWAS data from the updated FinnGen database for Mendelian randomization analysis. To enhance the reliability of causal associations, we conducted external validation and sensitivity analyses, including Bayesian colocalization and bidirectional Mendelian randomization analysis. Our study identified eight plasma proteins genetically associated with ILD or its subtypes. Among these, three proteins—CDH15 (Cadherin-15), LTBR (Lymphotoxin-beta receptor), and ADAM15 (A disintegrin and metalloproteinase 15)—emerged as priority biomarkers and potential therapeutic targets, demonstrating more reliable associations by passing a series of sensitivity analyses compared to the others. Based on these findings, we propose for the first time that CDH15, ADAM15, and LTBR hold promise as novel potential circulating protein biomarkers and therapeutic targets for the diagnosis and treatment of ILD, IPF, and sarcoidosis, respectively, especially ADAM15, and these findings have the potential to provide new perspectives for advancing the research on the heterogeneity of ILD.

## Introduction

Interstitial lung disease (ILD) encompasses a group of chronic, progressive disorders that primarily affect the lung interstitium and alveolar structures, characterized by diffuse inflammation, fibrosis, or a combination of both^1^. While these diseases may present with similar clinical, radiological, and pathological features, they often differ in etiology and disease progression^2,3^.

For example, idiopathic pulmonary fibrosis (IPF), a common subtype of ILD, is characterized by pathological changes of unknown etiology, unclear pathogenic mechanisms, and predominantly chronic progressive fibrosis^4^. This feature may be one of the key contributors to its poor prognosis, with a median survival of only 3 to 5 years^5–7^. Moreover, a growing number of case reports highlight that ILD is a common complication in patients with connective tissue diseases (CTDs), particularly systemic sclerosis (SSc)^8^. The risk of developing ILD is high during the first 5 years after SSc diagnosis and ILD is the leading cause of mortality in these patients^9^. Other ILD subtypes, including idiopathic nonspecific interstitial pneumonia (iNSIP) and hypersensitivity pneumonitis (HP), are also associated with poor prognoses^3^. In contrast, patients with sarcoidosis generally exhibit a more favorable overall prognosis than these other subtypes^10^.

Epidemiological data from the PERSEIDS study^11^, covering the years 2014–2018 and focusing on the European population, reveal an incidence of ILD ranging from 33.6–247.4 per 10^5^ persons, with IPF and systemic sclerosis-related ILD (SSc-ILD) ranging from 2.8–31.0 and 1.4–10.1 per 10^5^ persons, respectively. Another epidemiological investigation focused on sarcoidosis, which reported an annual incidence rate of 10–15 per 10^5^ persons in the European population^12^. Furthermore, fibrotic pathological changes predominate over inflammation, with fibrosing interstitial lung diseases (F-ILD) and non-IPF fibrosing ILD (non-IPF F-ILD) occurring at rates of 26.7–236.8 and 22.3–205.8 per 10^5^ persons. Sarcoidosis is the most common subtype of non-IPF F-ILDs. The data on progressive fibrosing ILD (PF-ILD) are particularly noteworthy, with an incidence ranging from 2.1 to 14.5/10^5^ person-years and a prevalence ranging from 6.9 to 78.0 per 10^5^ persons. Surprisingly, progressive fibrosis is also observed in one-third of non-IPF F-ILD cases. Even in sarcoidosis, approximately 10–30% of patients progress to progressive lung disease^13^. These findings support the emphasis of the 2022 ATS/ERS/JRS/ALAT clinical practice guidelines on recognizing progressive pulmonary fibrosis (PPF) as a phenotypic manifestation of specific ILDs rather than a distinct diagnosis, regardless of underlying diseases^14^. The prognosis may be similar to IPF when progression, usual interstitial pneumonia (UIP), or both are present in non-IPF fibrosing ILD (non-IPF F-ILD). Recent research on the trajectory of ILD has reported a higher incidence of progressive fibrosis (PF) behaviour occurring in up to 50% of ILD patients, which correlates with increased mortality rates^3^.

Based on these latest epidemiological data on ILD, with the extension of lifespan, it is foreseeable that the incidence of ILD and its subtypes, predominantly influenced by age, will continue to rise gradually. Furthermore, the number of patients with progressive fibrosis among non-IPF individuals is equally staggering, thus elevating non-IPF research to a status equivalent to IPF is urgently required. Unfortunately, the progress in the treatment of ILD remains discouraging. Due to the lack of curative treatments and therapies to improve symptoms, the prognosis and quality of life for patients with IPF and other subtypes of ILD, especially those with progressive disease, remain poor. However, hopes placed on ongoing ILD clinical trials in Phase II and III have not yet demonstrated any significant therapeutic effects^15–17^, and many studies have been halted or discontinued due to severe adverse reactions^18,19^. These challenges underscore the existing flaws in current drug development strategies for ILD, while recent advancements in human genetics offer promise as one of the potential solutions. Multiple studies demonstrate that drugs developed with support from human genetics evidence have at least twice the likelihood of approval^20,21^, and combining high-throughput, population-scale proteomics with human genetics for clearer causal gene mediation will undoubtedly increase this likelihood even further. Additionally, there is an increasing consensus in the research community that, given the heterogeneous nature of ILD, it is essential to carefully identify the specific characteristics of different clinical subgroups to advance more effective drug development and treatment strategies^2,22^.

Therefore, unlike previous studies focusing on IPF, this study stands out for its systematic analysis of diverse ILD subgroups, including IPF, sarcoidosis, and systemic autoimmune disease-associated ILD (SAD-ILD), and its utilization of up-to-date proteomic genetic data obtained through novel assays to identify therapeutic targets with greater specificity and efficacy.

## Methods

**Fig. 1:**
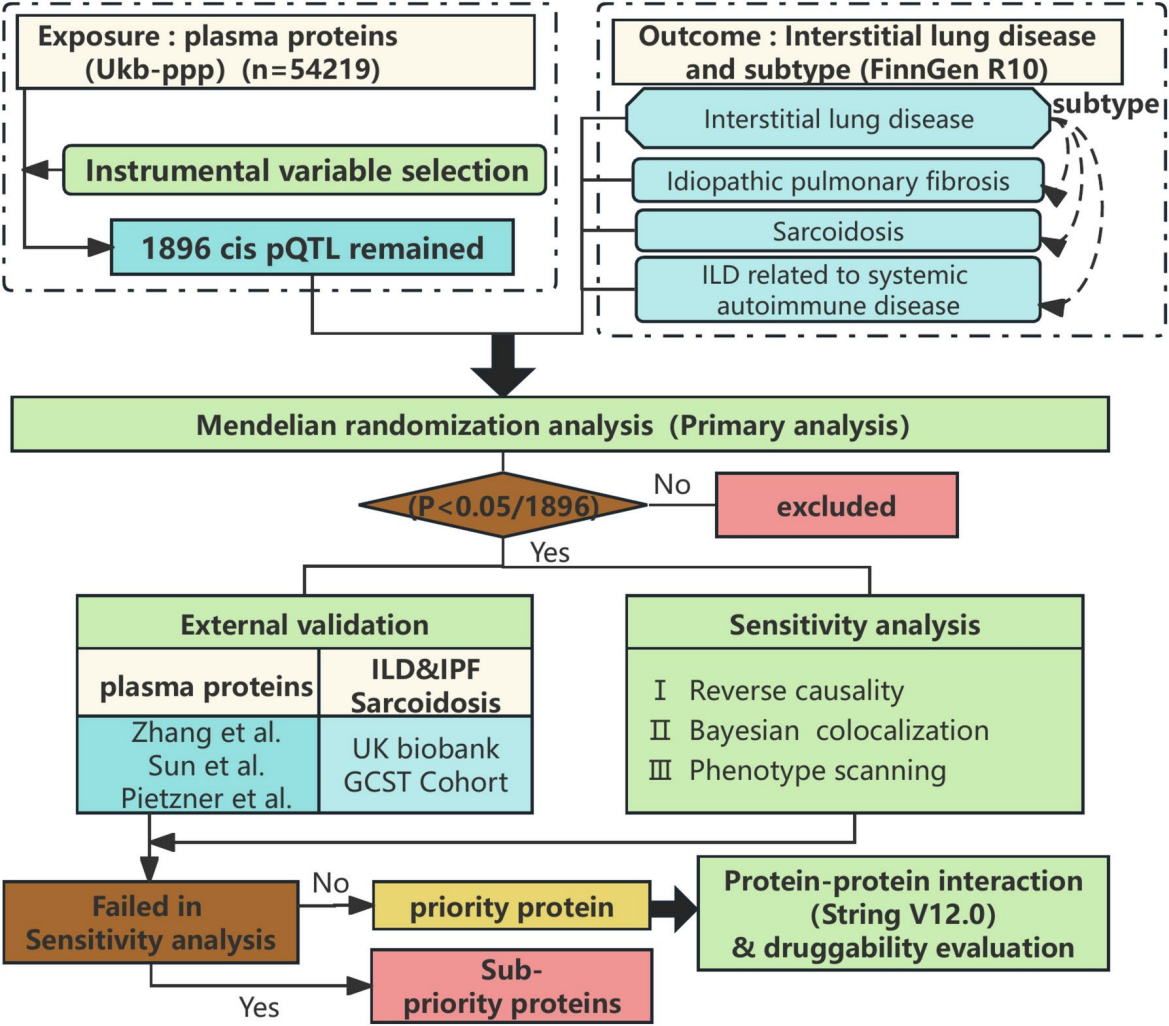
Study design for identification of plasma proteins causally associated with ILD and its subtypes.

Figure 1 outlines the overall study design. In detail, we utilized the latest large-scale antibody-based detection method for protein quantitative trait loci (pQTL) data from a European population and performed rigorous inclusion and exclusion screening on these data. We further examined their associations with ILD and its subtypes using a two-stage (discovery and replication) proteome-wide Mendelian randomization (MR) framework. The discovery phase refers to the initial MR analysis, which identifies potential causal associations. The replication phase involves performing MR analysis using independent GWAS datasets to validate and assess the reproducibility of the findings from the discovery phase. Given the potential for false-positive results in MR analyses, we employed a series of sensitivity tests—including Bayesian colocalization, bidirectional MR analysis, and phenome scanning—to ensure the identification of proteins with robust and reliable causal relationships established during the discovery phase. Finally, protein–protein interaction (PPI) and druggability evaluation of priority proteins were performed to explore the potential as therapeutic targets for ILD and its subtypes.

### Data source

We obtained genome-wide summary-level statistics for cis-pQTL from the UK Biobank Pharma Proteomics Project (UKB-PPP) study^23^. GWAS summary statistics for ILD and its subtypes can be retrieved from the GWAS catalog^24^, FinnGen (R10), and UK Biobank (UKB)^25,26^. Details of each GWAS were described in Supplementary Table S1. It is worth noting that the phenotypic characteristics of the sarcoidosis GWAS dataset used in this study do not specifically indicate that is sarcoidosis-associated ILD, which means that nearly half of the samples may not have progressed to ILD^27^. All GWASs were primarily based on the European population to ensure homogeneity of the study population. FinnGen (R10) had no sample overlap with the UK Biobank and GWAS catalog. Plasma pQTL data for MR external validation was available and obtained from the original studies^28–32^.

### Instrumental variable selection

The primary MR analysis in this study utilized plasma pQTL data from the latest large-scale proteogenomic study, the UKB-PPP study^23^, which utilized an antibody-based detection method to comprehensively localize pQTLs for 2,922 proteins among 54,219 participants, identifying 14,287 pQTLs at a multiple testing correction threshold of P < 1.7 × 10^–11^. To minimize bias caused by horizontal pleiotropy and linkage disequilibrium in Mendelian randomization analysis and to ensure the robustness of genetic instruments, we further screened the plasma pQTL data from the UKB-PPP study. Only pQTLs meeting the following criteria were included: (1) genome-wide significance level P < 5 × 10^–8^; (2) located outside the major histocompatibility complex (MHC) region (chr6, 26–34 Mb); (3) exhibiting independent associations (r^2^ < 0.001); (4) The pQTLs must be cis-acting, meaning they are located within 1 Mb of the transcription start site of the protein-coding gene. This criterion is based on the observation that genetic factors determining circulatory protein levels are typically located in cis (proximal) regions rather than trans (distal) single nucleotide polymorphisms (SNPs); (5) The F-statistic must exceed 30, ensuring the strength of the genetic instruments. Ultimately, the test identified 1896 cis-pQTLs for 1896 proteins (instrumental variables see Supplementary Table S2).

### Mendelian randomization analysis

In pursuing potential therapeutic targets for ILD and its subtypes, Mendelian randomization (MR) analysis aids in exploring the directionality from exposure to outcome in genetic studies. This study adhered strictly to three fundamental principles of Mendelian randomization analysis^33,34^: (1) Genetic variation must be reliably associated with exposure—Utilizing the most robust cis-pQTL variant associated with each plasma protein as instrumental exposure variable. (2) Genetic variation should not be associated with confounding factors of deterministic-outcome relationships—Enforcing strict inclusion criteria for instrumental variables and conducting multiple sensitivity analyses. (3) Genetic variation must not affect outcomes except through the benefits of exposure—Scrutinized via “Phenoscanner” and “LDlink”^35,36^. These principles ensure robustness and validity in identifying potential therapeutic targets through Mendelian randomization analysis.

In the primary MR analysis, we utilized summary statistical data from the FinnGen study (R10) of ILD and its subtypes as outcomes. The Wald ratio method from “TwoSampleMR” for proteins with a single cis-acting SNP to estimate the log odds change in ILD and its subtypes risk for per standard deviation (SD) increment or decrement of circulating protein levels as proxied by the instrumental variables^37,38^. In cases where a protein had two or more conditionally independent cis-pQTLs, considering the LD pattern among multiple cis-acting SNPs, the Inverse Variance Weighted (IVW) model was used to estimate MR effects and conduct pleiotropy and Cochran’s Q tests for heterogeneity examination^39^. In the preliminary analysis, stringent Bonferroni correction was applied to exclude multiple testing, using P < 0.05/1896 (P < 2.64 × 10^–5^) as the threshold to determine causality. Only proteins selected in the preliminary screening underwent MR external validation, with a replication MR P-value threshold set at 0.05, and outcome data sourced from the UK Biobank and GWAS catalog^40–42^. The same or significant variant strategy was employed, where the former used the same SNPs as genetic instruments as in the preliminary analysis, and the latter used the most significant pQTL for that protein from the study as instrumental tools to validate the primary findings.

### Reverse causality detection

To analyse the directionality of the causal association between the proteins preliminarily selected and ILD and its subtypes, and to detect potential reverse causal relationships, we applied filtering criteria of p < 5 × 10^–8^, r^2^ = 0.001, and clump_kb = 10,000 to extract instrumental variables for bidirectional MR analysis from the summary statistical data of FinnGen (R10), with Steiger test results as further supplementary validation.

### Bayesian colocalization analysis

Although MR serves as a powerful tool for detecting causal effects and employs cis-pQTL to minimize horizontal pleiotropy, linkage disequilibrium (LD) still may confound its results. In other words, positive results indicating a causal association between exposure and outcome might be false positives when influenced by different genetic variants. Therefore, to minimize the risk of such false positives, we conducted a Bayesian colocalization analysis through the coloc.abf algorithm to assess the potential LD between the proteins preliminarily identified and ILD and its subtypes^43^.

This analysis method utilizes the “coloc” package with default parameters (p1 = 1 × 10^−4^, p2 = 1 × 10^−4^, and p12 = 1 × 10^−5^) to evaluate the probability of shared causal variants between the protein and trait^44^. The “Locuscomparer” package was used to visualize the region results of colocalization^45^. In this study, we primarily focused on the posterior probability of hypothesis 4 (PPH_4_), which indicates strong evidence if PPH_4_ > 0.8 that the protein and trait share the same causal variant within the genomic locus^36^.

### Phenotype scanning

To minimize the potential for horizontal pleiotropy in instrumental genetic variables, we conducted phenotype scans using “Phenoscanner” and “LDlink”^35,36^. SNPs were considered pleiotropic if they met the following criteria: (1) The association is significant at the genome-wide level (P < 5 × 10^–8^). (2) The SNP is associated with known risk factors for ILD and its subtypes, including metabolic traits, proteins, or clinical characteristics.

### PPI and druggability evaluation on the potentials of therapeutic targets

Furthermore, to explore whether potential drug targets for ILD and its subtypes are strongly associated with existing therapeutic drugs on the market and to unearth promising drug targets, we conducted a PPI analysis using the STRING database (V12.0). We set the minimum required interaction score threshold to 0.7. Additionally, we searched the PubMed database for literature evidence concerning these promising drug targets to assess their feasibility and credibility as drug targets.

## Results

### Screening the proteome for ILD causal proteins

**Fig. 2:**
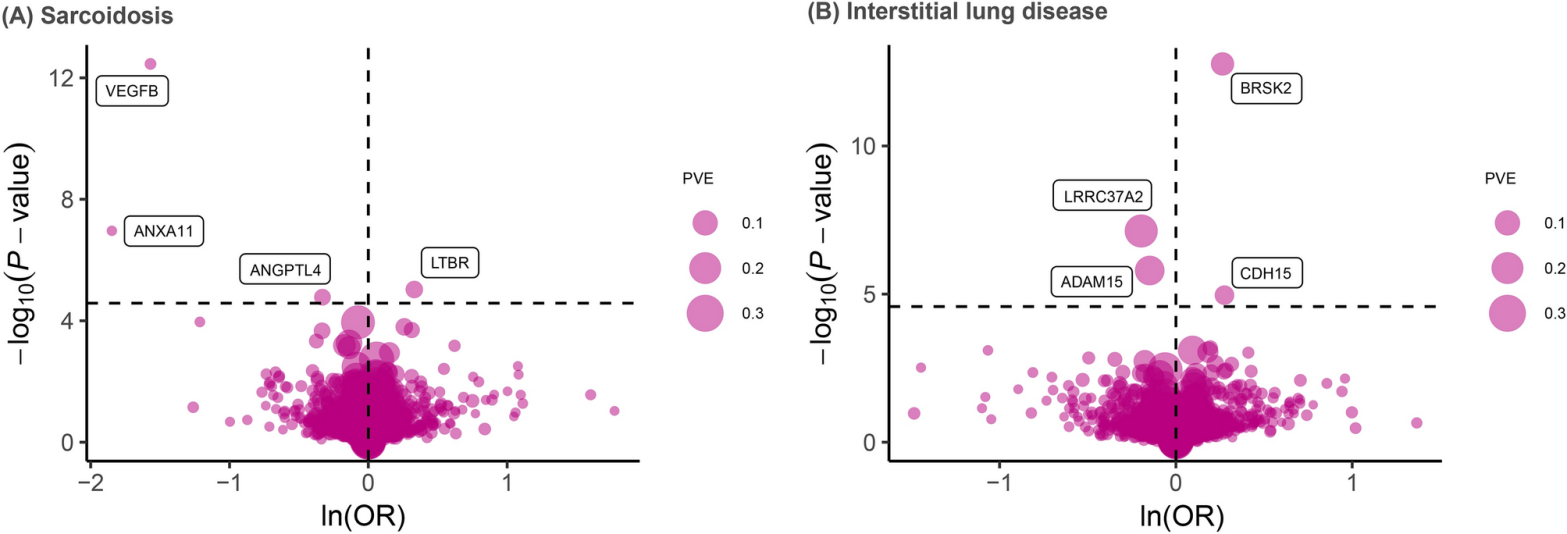
Volcano plots of the MR results for proteins on the risk of (**A**) Sarcoidosis and (**B**) Interstitial lung disease. OR for increased risk of ILD and Sarcoidosis were expressed as per SD increase in plasma protein levels. The dashed horizontal black line corresponded to P = 2.64 × 10^–5^ (0.05/1896). ln = natural logarithm; PVE = proportion of variance explained.

After applying Bonferroni correction (P < 2.64 × 10⁻^5^), our primary Mendelian randomization (MR) analysis identified potential causal relationships between eight proteins and ILD or its subtypes. Specifically, four plasma proteins showed significant associations with ILD, with similar associations observed in IPF. The Wald ratio indicated that the genetically predicted increase in CDH15(Cadherin-15) was associated with an elevated risk of ILD, with an odds ratio (OR) of 1.32 (95%CI 1.16–1.49; P = 1.09 × 10^−5^). Similarly, elevating the expression levels of BRSK2(Serine/threonine-protein kinase BRSK2) (OR = 1.30; P = 1.70 × 10^−13^) also increased the risk of ILD, while increasing the expression levels of ADAM15(A disintegrin and metalloproteinase 15) (OR = 0.86, 95% CI 0.81–0.92; P = 1.59 × 10^−6^) and LRRC37A2(Leucine-rich repeat-containing protein37A2) (OR = 0.82; P = 7.40 × 10^−8^) decreased the risk of ILD. Similarly, the odds ratio (95% CI) of IPF per standard deviation increase in genetically predicted levels of protein was 1.40 (1.26–1.55) for BRSK2, whereas 0.81 (0.75–0.89) for ADAM15, 0.74 (0.66–0.82) for LRRC37A2. Notably, BRSK2, LRRC37A2, and ADAM15 exhibited shared associations, while CDH15 showed no significant association with IPF (refer to Table 1 and Fig. 2).

**Table 1:** Summary of potential causal proteins for ILD and IPF.

Protein	SNP	Idiopathic pulmonary fibrosis (IPF)					Interstitial lung disease (ILD)					Steiger filtering
		Primary MR analysis		Bidirectional MR(IVW)		coloc.abf (PPH4)	Primary MR analysis		Bidirectional MR(IVW)		coloc.abf (PPH4)	
		OR (95% CI)^a^	P-value	P- value	β-IVW		OR (95% CI)^a^	P-value	P-value	β-IVW		
ADAM15	rs11589479	0.81 (0.75,0.89)	5.25 × 10^−6^	0.759	﻿− 0.003	0.966	0.86(0.81,0.92)	1.59 × 10^−6^	0.781	− 0.004	0.997	Passed (2.89 × 10^**–**15^)
CDH15	rs11646135	1.39(1.16,1.65)	2.49 × 10^−4^	0.197	0.009	0.796	1.32(1.16,1.49)	1.09 × 10^−5^	0.516	0.006	0.863	Passed (1.85 × 10^–22^)
LRRC37A2	rs62057151	0.74 (0.66,0.82)	1.73 × 10^−8^	0.413	− 0.124	0.543	0.82(0.76,0.88)	7.40 × 10^−8^	0.514	− 0.168	0.419	False (2.00 × 10^–55^)
BRSK2	rs7395567	1.40 (1.26,1.55)	6.41 × 10^−11^	0.003	0.083	6.83 × 10^–147^	1.30(1.21,1.40)	1.70 × 10^−13^	2.28 × 10^–4^	0.145	5.01 × 10^–156^	False (1.47 × 10^–2^)
FUT3_FUT5	rs3760775	0.95 (0.87,1.04)	0.259	–	–	–	0.96 (0.90,1.03)	0.248	–	–	–	–

^a^Odds ratios for increased risk of Interstitial lung disease or its subtypes were expressed as per SD increase in plasma protein levels.

In contrast, the results for sarcoidosis identified four candidate proteins that were entirely distinct from those associated with ILD and IPF after Benjamini–Hochberg correction. Specifically, higher plasma levels of LTBR(Lymphotoxin-beta receptor) were associated with a significantly increased risk of developing sarcoidosis, with on average 39% increased risk(OR = 1.39; p = 9.38 × 10^−6^), while ANGPTL4(Angiopoietin-related protein 4)(OR = 0.72; p = 1.68 × 10^–5^), VEGFB(Vascular endothelial growth factor B) (OR = 0.21; p = 3.49 × 10^–13^) and ANXA11(Annexin A11) (OR = 0.16; p = 1.09 × 10^–7^) were associated with decreased risk, respectively(refer to Table 2 and Fig. 2). Thus, we will further subject the above proteins to a series of sensitivity tests to identify the most reliable ones.

**Table 2:** Summary of potential causal proteins for Sarcoidosis.

Protein		SNP		Primary MR analysis				Bidirectional MR(IVW)		coloc.abf (PPH4)		Steiger filtering		Previously reported associations	
				OR (95% CI)^a^		P-value		P-value	β-IVW						
VEGFB		rs660442		0.21(0.14,0.32)		3.49 × 10^−13^		0.733	− 0.010	0.907		Passed (1.93 × 10^–6^)		Rheumatoid arthritis	
ANXA11		rs2784773		0.16(0.08,0.31)		1.09 × 10^−7^		0.303	− 0.022	8.47 × 10^–6^		Passed (1.58 × 10^–9^)		Sarcoidosis	
LTBR		rs10849449		1.39(1.20,1.61)		9.38 × 10^−6^		0.637	0.006	0.947		Passed (9.88 × 10^–18^)		Chronic obstructive pulmonary disease liability	
ANGPTL4		rs2278236		0.72(0.62,0.84)		1.68 × 10^−5^		0.016	− 0.033	0.957		Passed (1.61 × 10^–13^)		High-density lipoprotein cholesterol levels	

^a^Odds ratios for increased risk of Interstitial lung disease or its subtypes were expressed as per SD increase in plasma protein levels.

Furthermore, unfortunately no significant association was found between SAD-ILD and any proteins in the study. And for exploring specific circulating proteins associated with non-IPF F-ILD and other non-IPF ILD subtypes, we analyzed datasets categorized as “Other interstitial pulmonary diseases with fibrosis” and “Other interstitial pulmonary diseases” from the UK Biobank and GWAS Catalog for MR analysis. However, these analyses did not yield any significant results. Additionally, some previously described biomarkers for IPF^46^, specifically FUT3_FUT5, were also evaluated in this MR study (the protein quantification method in the UKB-PPP study categorized FUT3 and FUT5 under the same cis-pQTL) (refer to Table 1).

### External validation of potential drug targets

**Fig. 3:**
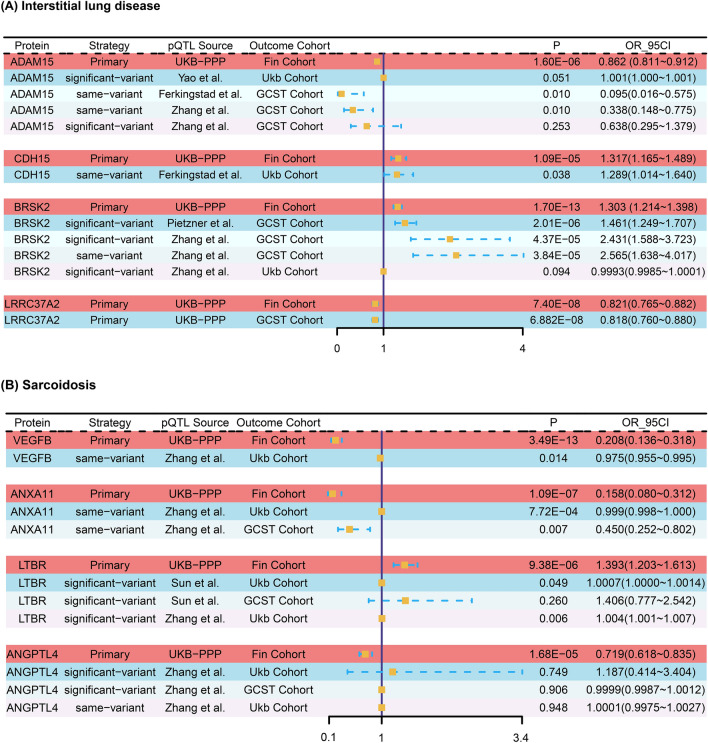
External validation of the causal relationship between potential causal proteins and (**A**) Interstitial lung disease or (**B**) Sarcoidosis.

To replicate the preliminary findings across various outcome GWAS datasets, we adopted both the same-variant and significant-variant approaches (refer to Supplementary Table S3). Notably, significant associations for BRSK2 were identified in both ILD and IPF GWAS Catalog cohorts using two strategies. Specifically, employing independent significant pQTL documented by Zhang et al. as genetic instruments^28^, elevated expression of BRSK2 was associated with an increased risk of ILD(OR = 2.43; 95%CI 1.59–3.72; p = 4.37 × 10^–5^). For ADAM15 and CDH15, significance was only in the ILD GWAS Catalog cohort using the same-variant strategy (P < 0.05). As no other independent significant pQTLs were for LRRC37A2, the original pQTL continued, still significantly associated in both external cohorts for ILD and IPF. Furthermore, LTBR showed significant associations in the sarcoidosis UK Biobank cohort using the significant-variant strategy, while VEGFB and ANXA11 showed associations using the same-variant strategy. However, ANGPTL4 did not yield notable results upon external validation (refer to Fig. 3).

The figures visually show the differences for specific proteins in OR and 95%CI between the primary and replicated analyses. Therefore, we conducted heterogeneity tests separately for the MR analysis results of each protein and found that almost all exhibited high heterogeneity (I^2^ > 75%) (refer to Supplementary Figure S1). Given the larger sample size and higher credibility of evidence from the primary analysis, we relied on it for the risk ratio of proteins to the disease.

### Sensitivity analysis for causal proteins

Firstly, in the bidirectional MR analysis, we observed a genetically predicted significant inverse causal association between ILD and IPF and BRSK2(ILD: β_IVW_ = 0.145, p = 2.28 × 10^–4^; IPF: β_IVW_ = 0.083, p = 2.56 × 10^–3^), Further Q-tests and pleiotropy tests revealed evidence of heterogeneity (IPF: p_Q_ = 1.15 × 10^–27^, p_pleiotropy_ = 0.01; ILD: p_Q_ = 1.15 × 10^–18^, p_pleiotropy_ = 0.03). Leave-one-out analysis indicated that rs35705950, identified via LDlink as a robust genetic instrument for ILD and IPF and lacking a direct association with BRSK, was the primary driver of this observed effect. After removing this genetic instrument variable, no reverse causal association or heterogeneity was observed. In addition to BRSK2, sarcoidosis also demonstrated a reverse causal association with ANGPTL4 (β_IVW_ = -0.03, p = 0.016), and the absence of heterogeneity (p_Q_ = 0.12, p_pleiotropy_ = 0.86) further reduced the likelihood of false-positive results and suggested that this reverse causal effect was not contributed by individual SNP. Furthermore, ILD or its subtypes had no observed causal effects on the remaining six proteins. Steiger filtering further strengthened the evidence for the directionality of the associations (refer to Tables 1, 2, Supplementary Table S4 and Supplementary Figures S2–S3).

**Fig. 4:**
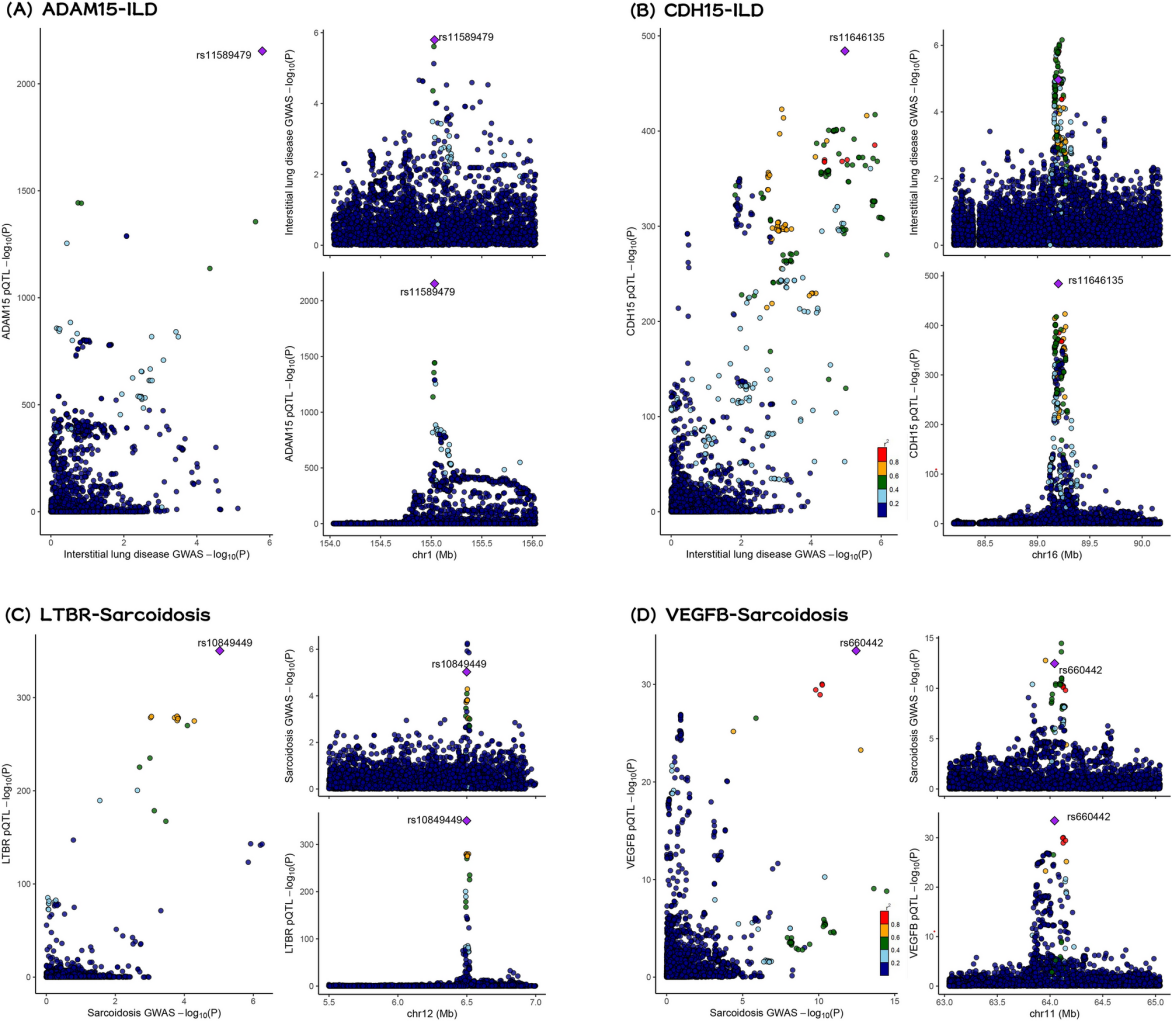
Bayesian colocalization analysis of (**A**) ADAM15-ILD, (**B**) CDH15-ILD, (**C**) LTBR-Sarcoidosis and (**D**) VEGFB-Sarcoidosis. Diamond purple points represented the SNP that with the minimal sum of P value in corresponded protein GWAS and ILD or its subtypes GWAS.

Secondly, Bayesian colocalization based on pQTLs strongly indicated that ADAM15 (coloc.abf-PPH_4_ = 0.997) and CDH15 (coloc.abf-PPH_4_ = 0.863) share the same variants with ILD, and IPF also shares the same variants with ADAM15 (coloc.abf-PPH_4_ = 0.966). Additionally, three out of the four proteins preliminarily identified for sarcoidosis—VEGFB (coloc.abf-PPH_4_ = 0.907), LTBR (coloc.abf-PPH_4_ = 0.947), and ANGPTL4 (coloc.abf-PPH_4_ = 0.957)—received robust support by genetic colocalization analysis (PPH_4_ > 0.8) under standard priors and window (± 1 Mb). On the other hand, BRSK2, LRRC37A2, and ANXA11 did not share the same variants with ILD or its subtypes, suggesting that the causal associations of these proteins were likely driven by different SNPs within their respective genomic regions (refer to Tables 1, 2, Fig. 4 and Supplementary Figure S4). This phenomenon highlighted the possibility of LD confounding even with cis-pQTL.

Lastly, to mitigate bias from horizontal pleiotropy, we utilized “Phenoscanner” and “LDlink” to identify significant associations (P < 5 × 10^–8^) between rs660442 and rs2278236—cis-pQTLs for VEGFB and ANGPTL4, respectively—and phenotypes potentially linked to sarcoidosis. Specifically, our analysis through LDlink found that rs660442 is strongly associated with rheumatoid arthritis (RA). Previous epidemiological studies have shown a higher prevalence of sarcoidosis co-occurring with RA and other autoimmunity diseases compared to normal controls^47^, which is related to sarcoidosis being classified as a specific autoimmune disease and the typical characteristic of clustering among autoimmune diseases^48,49^. Additionally, literature suggests that HLA-DR4 may serve as a common genetic risk factor for the co-occurrence of sarcoidosis and RA^50^, indicating a shared genetic basis for their comorbidity. Consequently, we cannot rule out the possibility that RA might enhance the causal effect of VEGFB on sarcoidosis. Moreover, rs2278236 is associated with high-density lipoprotein (HDL) cholesterol levels. Prior epidemiological and genetic studies have reported significant genetic overlap between lipid metabolism and immune-related disorders^51^. Similarly, this overlap raises the possibility that confounding factors might contribute to false-positive findings. Beyond these specific observations, we did not identify any alternative genetic instruments, such as ADAM15 (rs11589479), that are associated with known risk factors for ILD or its subtypes (refer to Tables 1 and 2).

Based on the evidence outlined above, we categorized the eight preliminary identified proteins into priority and sub-priority groups according to their performance in sensitivity analyses, specifically regarding the reliability of their causal associations with ILD and its subtypes. Three proteins (ADAM15, CDH15 and LTBR) passed all tests, as they did not show linkage disequilibrium in the colocalization analysis (PPH_4_ > 0.8), had no reverse causal associations, and the causal effects indicated by genetic instruments were not confounded by confounding factors, making them priority candidates for further exploration as plasma protein markers and potential drug targets. Proteins that passed colocalization analysis but failed other sensitivity analyses, such as ANGPTL4 and VEGFB, or proteins that failed colocalization analysis, such as LRRC37A2, BRSK2, and ANXA11, showed poor reliability in their causal associations and are classified as sub-priority.

### PPI and druggability evaluation on the potentials of therapeutic targets

The PPI network suggests potential associations of the priority proteins (refer to Supplementary Table S5). Specifically, using STRING (Version12.0), a tool that evaluates the degree of association between proteins based on text mining, co-expression, experimental evidence, and other methods, we found that CDH1, associated with ADAM15, and CDH2, associated with CDH15, were closely related in previous studies related to pulmonary fibrosis.

We attempted to discover a causal association between ADAM15 and CDH15 through single-sample MR analysis using GWAS data from these proteins in the UKB-PPP dataset. We found that ADAM15 has a causal association with CDH15(IVW: p = 0.043) , while the reverse is invalid. The MR-Egger intercept did not significantly deviate from zero in our study (p_pleiotropy_ = 0.444) (refer to Supplementary Figure S5), suggesting no evidence of horizontal pleiotropy. Additionally, ADAM15 showed bidirectional causal associations with CDH1 (IVW: p < 0.05). These findings suggest potential causal associations between these proteins at the plasma level, and in particular the unidirectional causal association of ADAM15 with CDH15 is of interest. Due to the presence of heterogeneity (p_Q_ < 0.05), we used IVW’s random-effects model to minimize the effect on the results (refer to Supplementary Table S6 and Supplementary Figures S5-S6).

Additionally, we discovered reliable interactions between ADAM15 and Src tyrosine kinase (SRC), HCK, and CDH15 and CTNNB1 (with a minimum required interaction score threshold > 0.7). Some of these proteins have been previously identified as targets for current treatments of ILD and IPF. Specifically, SRC is one of the targets for Nintedanib^52^, a drug recommended in the current IPF treatment guidelines. Furthermore, the mechanism by which increased expression levels of CDH1 delay the progression of pulmonary fibrosis may be explained by the targeted regulation of YTHDF2 by miR-494-3p to delay the epithelial-mesenchymal transition (EMT)^53^. Additionally, studies have reported that the anti-fibrotic function of CDH15 with the NRF2 activator sulforaphane (SFN) may be achieved by inhibiting the expression of CDH2^54^. These findings suggest substantial evidence demonstrating a potential association and feasibility of ADAM15 and CDH15 with ILD in terms of molecular mechanisms and drug development. Furthermore, for LTBR, we found that it has the strongest correlation with LTB.

## Discussion

To explore potential drug targets and circulating protein biomarkers specific to ILD or its subtypes, we comprehensively collected relevant datasets. Eight circulating proteins were identified to have significant causal associations with ILD or its subtypes in the primary MR analysis. Through rigorous sensitivity analyses, we ultimately identified three proteins as priority candidates with significant druggable potential: CDH15 for ILD excluding IPF, ADAM15 for both IPF and ILD, and LTBR for sarcoidosis.

Notably, whether ADAM15 serves as a specific biomarker for IPF or is broadly present in patients with various ILD subtypes, as well as which specific ILD subtypes (excluding IPF and sarcoidosis) CDH15 may target, remains to be determined through the discovery of more detailed GWAS datasets for ILD subtypes. Given the clinical need for simple and minimally invasive auxiliary diagnostic methods to distinguish ILD subtypes, this observation is particularly significant. Furthermore, we found that the causal proteins of sarcoidosis are completely different from those of ILD and IPF. It undoubtedly provided evidence of heterogeneity in circulating protein levels between the sarcoidosis population and common subtypes of ILD, including IPF. Combining epidemiological data suggested that sarcoidosis accounted for a considerably small proportion of the ILD dataset compared to IPF.

Additionally, we conducted a comprehensive literature search and found that our preliminary analysis failed to replicate findings from previous studies focused on IPF^46^. This discrepancy could be attributed to differences in the methods used to detect pQTL. Previous studies utilized proteomic genetic data based on aptamer-based detection methods, while this study employs antibody-based genetic research proteomic data. Comparative assessments indicate that the aptamer-based detection method offers broader biological coverage and higher accuracy, whereas the antibody-based detection method exhibits greater protein target specificity, with stronger correlations to certain diseases and immune assays^55^. Thus, researchers widely accept that these two technologies, capturing different features of protein chemistry, serve as complementary tools for biomarker discovery^56^. At the same time, differences in the datasets may be one of the reasons. Compared to previous studies, the dataset used in this study had a significantly larger sample size, which may make the results of this study more convincing. Notably, among the eight circulating proteins identified in this study as potentially causally related, six had not been previously reported in the literature as directly associated with ILD or its subtypes. This suggests that we may have identified new circulating proteins closely linked to ILD or its subtypes. These proteins include ADAM15, CDH15, LRRC37A2, BRSK2, ANGPTL4, and VEGFB. While ANXA11, which has been widely reported to be associated with genetic polymorphisms in sarcoidosis^57^, was further supported by our study at the proteomic level.

Given the priority of ADAM15, CDH15, and LTBR, we primarily conducted pharmacological assessments on these newly identified potential drug targets. ADAM15 is a transmembrane-anchored multi-domain protein belonging to the disintegrin metalloproteinase family^58^. Increasing evidence suggests that Src family kinases (SFKs), including SRC and HCK, which are strongly correlated with ADAM15, are involved in the pathogenesis of pulmonary fibrosis. For instance, Src kinase can promote fibrosis by mediating fibroblast adhesion and invasion^59,60^, facilitating alveolar bronchialization^61^, inducing capsule formation^62^, and promoting extracellular matrix (ECM) deposition^52^. The current standard treatment for IPF, Nintedanib, acts as a specific Src kinase inhibitor, lending support to the reliability of the priority proteins identified in this study. Moreover, the effects of other specific Src kinase inhibitors—Bosutinib and Saracatinib—have been validated in numerous in vitro and in vivo experiments^63,64^, showing signs of efficacy that may exceed that of Nintedanib in some studies. ADAM15 has been found to regulate SRC in the inflammatory synovial tissue of RA, which can convert the apoptosis signal induced by FasL into triggering SRC/FAK phosphorylation to resist fibroblast apoptosis^65,66^. One of the characteristics of IPF is the abnormal accumulation of fibroblasts^67^. Therefore, whether similar mechanisms occur in interstitial lung disease fibrosis or whether they are one of the mechanisms of Src kinase inhibition is worthy of further investigation. In addition, CDH1 strongly associated with ADAM15, and CDH2 strongly associated with CDH15 have also been found to play significant roles in IPF. CDH1 and CDH2 are both calcium-dependent cell adhesion proteins^67^. However, whereas CDH1 promotes cell–cell adhesion and is a crucial molecule for maintaining epithelial cell phenotype^68^, CDH2 acts oppositely. Numerous studies indicate that a hallmark of one of the primary pathological mechanisms in IPF, epithelial-mesenchymal transition (EMT), is the loss of E-cadherin (E-cad, encoded by CDH1 mRNA, an epithelial marker), along with increased expression of Vimentin (a mesenchymal marker) and N-cadherin (N-cad, encoded by CDH2 mRNA)^69–71^. In this regard, targeting YTHDF2 with miR-494-3p can enhance the expression level of CDH1^52^. On the other hand, the NRF2 activator sulforaphane (SFN) can inhibit the expression of CDH2^54^, thus exerting anti-fibrotic functions. In recent years, some studies have been conducted on the anti-fibrotic mechanisms of miR-494-3p targeting CDH1 and the NRF2 activator sulforaphane (SFN) targeting CDH2^72^. However, there is still a relative scarcity of animal and cell-based research specifically focused on ILD, and comparative studies involving in vivo experiments with the current standard-of-care (SoC) drugs for IPF are highly anticipated. Our study also established a close association between ADAM15 and the CDH family through MR analysis, specifically the positive causal association of ADAM15 with CDH15. Given the limited studies directly linking ADAM15 and CDH15 to ILD and IPF, we propose a novel hypothesis that ADAM15 may occupy an upstream position in the ILD cascade. It could exert its effects by regulating the expression of CDH family members, such as CDH15, CDH1, and CDH2, or phosphorylating the Src kinase family. Future studies should focus on and test this hypothesis.

LTBR is a unique receptor for LTB and belongs to the tumor necrosis factor (TNF) ligand family. Previous reports have shown significantly increased expression of LTB in major chronic inflammatory diseases characterized by lymphocytic infiltration, such as sarcoidosis^73^. An intriguing finding previously reported is that LTBR is exclusively present in the epithelial cells lining the bronchial lumen, while its homolog, LTB, is predominantly distributed in granulomas and surrounding lymphocytes^74^. Our study found that elevated circulating levels of LTBR lead to increased susceptibility to sarcoidosis. These associative findings are worth further investigation in the future. In summary, whether LTBR holds promise as a circulating biomarker for aiding in the differential diagnosis of sarcoidosis requires further investigation. At the same time, LTB may also play a role in the sustained inflammation observed in sarcoidosis granulomas, making it a potential therapeutic target.

Like other Mendelian randomization (MR) studies, our approach may also have inevitable limitations. MR results may be biased due to potential violations of its assumptions, such as the complex interplay between genetics and environment. However, our study design aimed to minimize explicit biases by using cis-pQTL to reduce potential horizontal pleiotropy and conducting rigorous sensitivity analyses to assess potential pleiotropic effects, excluding any initial screening proteins that did not meet these criteria. Additionally, we found high heterogeneity in the majority of the externally validated results. We attribute this to the difference in the datasets and methods of detective pQTL used in the primary and replication analyses. Given the current predominance of proteomic genetic data derived from the aptamer detection method and the scarcity of large-scale antibody-based detection methods, this hypothesis could not be thoroughly validated. The observed heterogeneity might also stem from the substantial diversity among ILD disease subtypes, leading to the inclusion of disparate populations across different datasets. At the same time, the lack of datasets for various ILD subtypes, especially iNSIP and CTD-ILD, and the small sample sizes in datasets such as SAD-ILD and non-IPF F-ILD limited our study results. Furthermore, we believe that utilizing a GWAS dataset specifically characterized by sarcoidosis-associated ILD would be more appropriate than a dataset reflecting general sarcoidosis phenotypes, as this implies that nearly half of the samples may not have progressed to ILD, and isolating cases of sarcoidosis with confirmed ILD involvement could yield greater specificity in analysis. Unfortunately, we have not yet identified a suitable dataset to replace the current one. As future studies incorporate more samples representing diverse ILD subtypes, refined subtype-specific GWAS can address these issues and enable more precise Mendelian randomization analyses. Such efforts are pivotal for advancing precision medicine, intending to optimize the efficacy of future therapies through targeted therapy. Lastly, research on ILD among non-European ethnic populations was limited, underscoring the need for further investigations to ascertain the generalizability of these findings.

## Conclusion

In conclusion, by leveraging the latest characterization of genetic structure in plasma proteomics and conducting Mendelian randomization (MR) analyses on interstitial lung disease (ILD) and its various subtypes, supplemented by comprehensive assessments including Bayesian colocalization, we identified several circulating proteins with causal associations to ILD and its subtypes. Among these, ADAM15, CDH15, and LTBR emerged as priority biomarkers and potential therapeutic targets, with a particular emphasis on ADAM15. However, given the complexities of drug development and the disease itself, as well as the limitations of MR analysis, we believe the value of MR analysis lies in its ability to screen for reliable and promising drug targets cost-effectively but further experimental studies are warranted to evaluate the true efficacy of these candidate proteins.

## Supplementary Information

Supplementary Information 1.

Supplementary Information 2.

Supplementary Information 3.

## Data Availability

The datasets analyzed during the current study are available at the GWAS catalog(https://www.ebi.ac.uk/gwas/), FinnGen(https://www.finngen.fi/en/) and UK Biobank (http://www.nealelab.is/uk-biobank). Additionally, the original studies for plasma pQTL with the PMID were listed in Supplementary Table S1.
