# Supplementary material for: Proteome-wide mendelian randomization identifies causal plasma proteins in interstitial lung disease
Source: Sci Rep. 2025 Jan 17;15:2293. doi: 10.1038/s41598-025-85338-y (PMC11748740; doi:10.1038/s41598-025-85338-y)
Supplement: Supplementary file 2 — Supplementary Information 2. [file 41598_2025_85338_MOESM2_ESM.docx]

**Fig. S1 Heterogeneity test of MR analysis results for each potential causal protein with ILD or its subtypes**

1. **ADAM15- Interstitial lung disease**


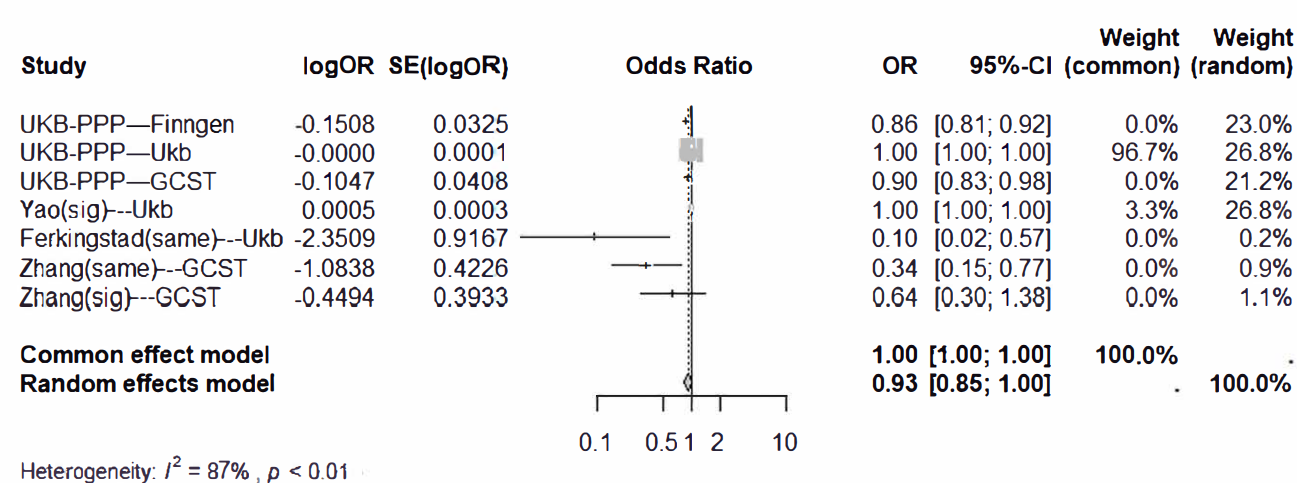


1. **BRSK2 - Interstitial lung disease**


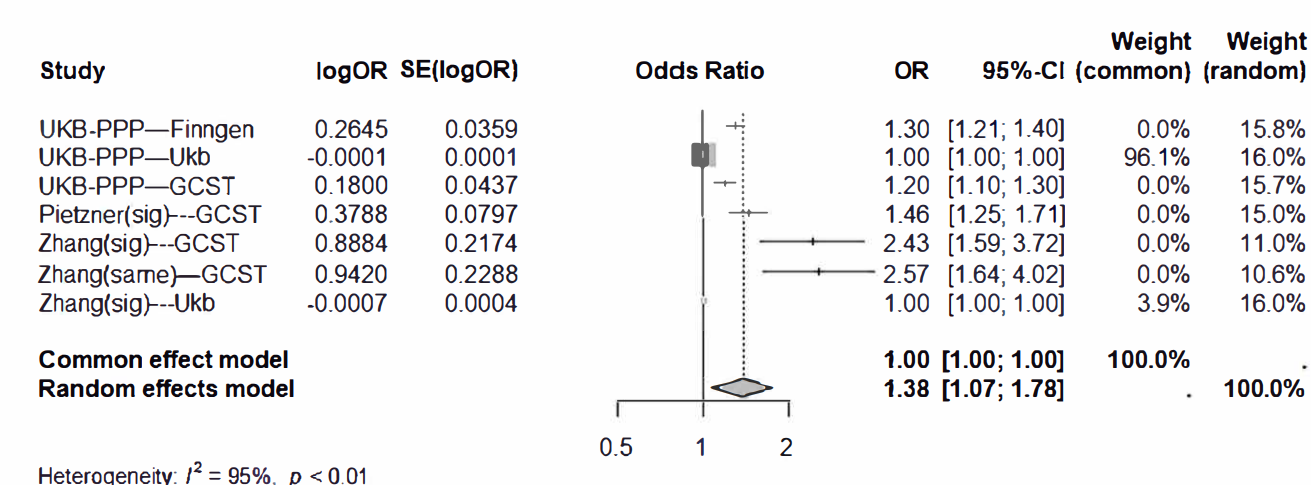


1. **BRSK2 -** **Idiopathic pulmonary fibrosis**


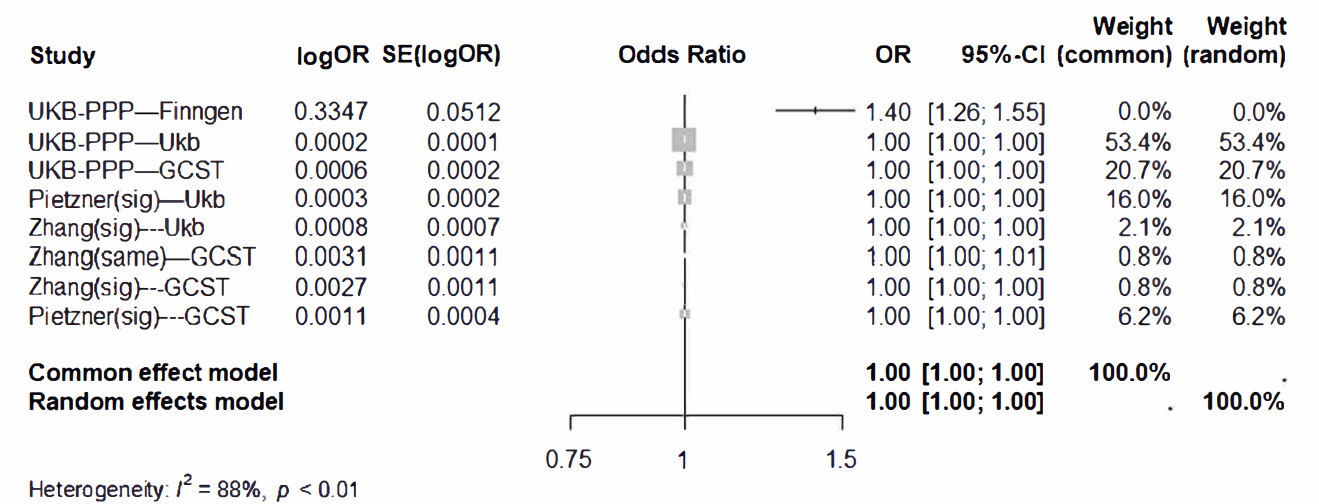


1. **LTBR-Sarcoidosis**


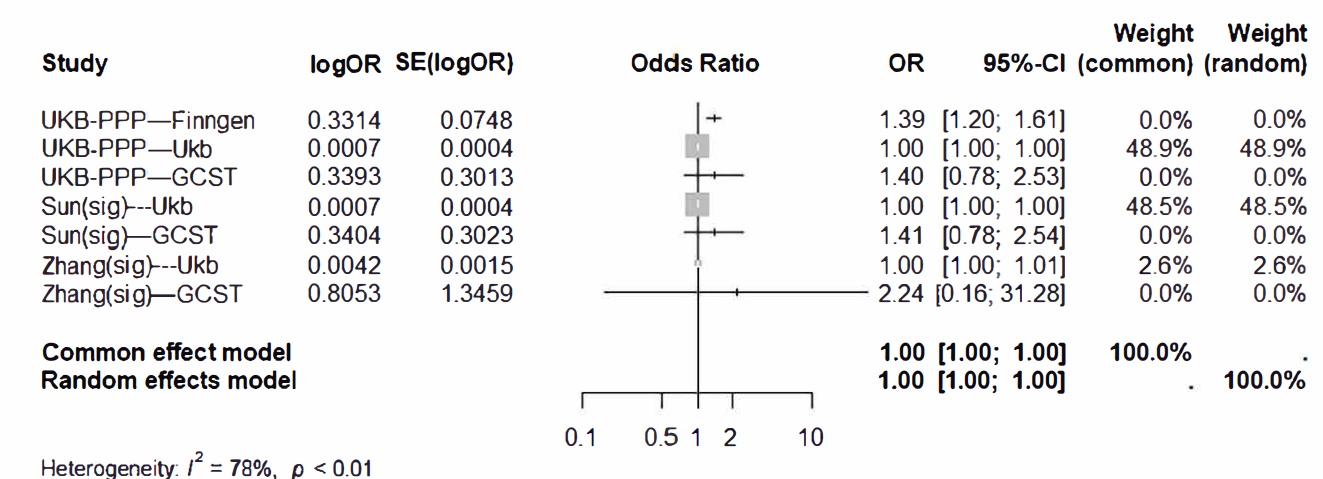


Odds ratios for increased risk of Interstitial lung disease or its subtypes were expressed as per SD increase in plasma protein levels

The range of I² values is from 0 to 100%, indicating the degree of heterogeneity among study results. An I² of 0 indicates no heterogeneity among all study results, meaning the results are entirely consistent; an I² of 100% indicates extreme heterogeneity among all study results, meaning there is a significant difference in results; an I² value between 25% and 75% is considered moderate to high heterogeneity.

**Fig.S2 The heterogeneity and pleiotropy analysis of potential causal proteins inversely associated with ILD or its subtypes in bidirectional Mendelian randomization.**

1. **Scatter plot of interstitial lung disease and BRSK2**


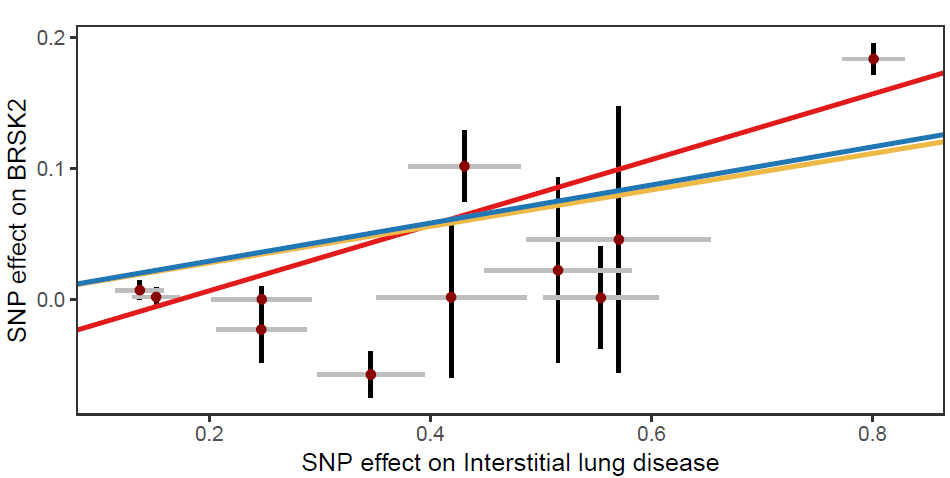

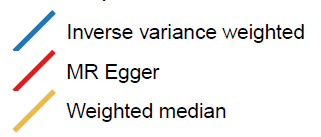


1. **Leave-one-out plot of interstitial lung disease and BRSK2**

**
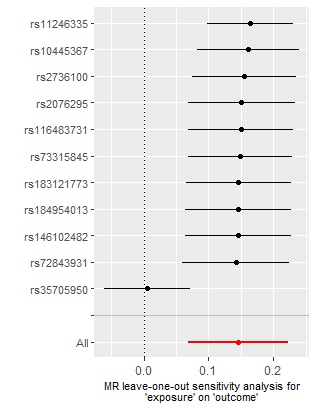
**

1. **Scatter plot of interstitial lung disease and BRSK2 (removal of rs35705950)**

**
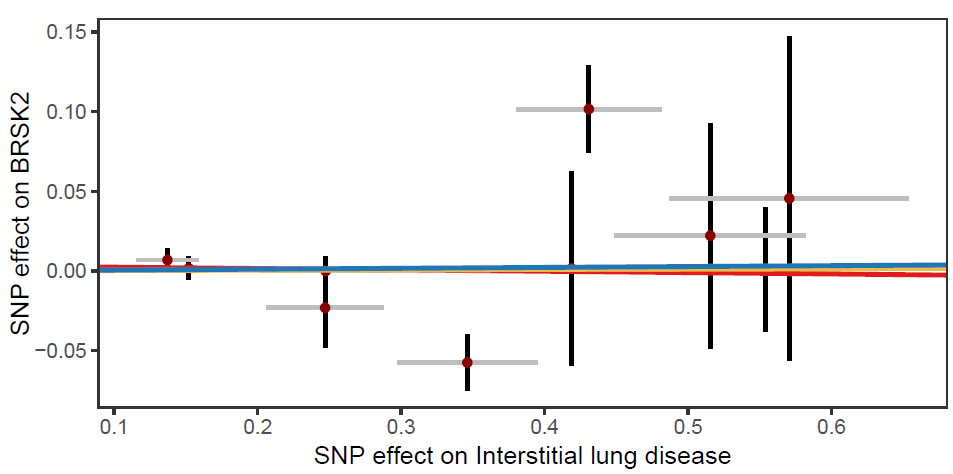
**
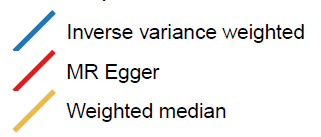


1. **Leave-one-out plot of interstitial lung disease and BRSK2 (removal of rs35705950)**

**
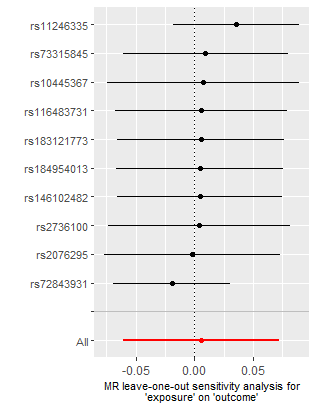
**

1. **Scatter plot of sarcoidosis and ANGPTL4**


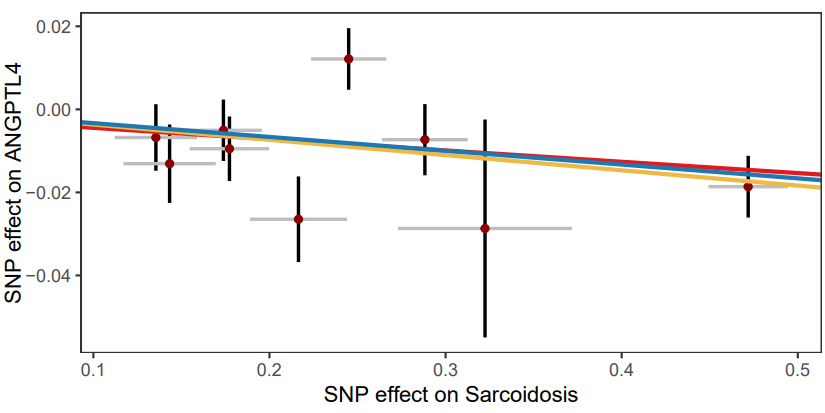

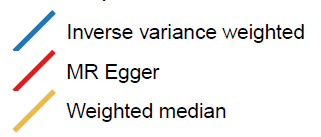


1. **Leave-one-out plot of sarcoidosis and ANGPTL4**


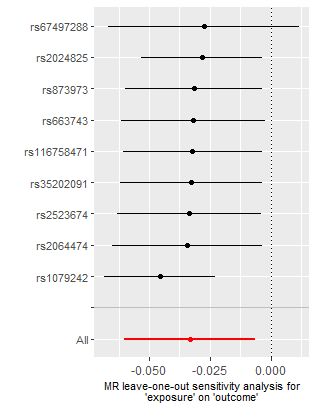


Scatter plot to visualize causal effect of interstitial lung disease or sarcoidosis on potential causal proteins. The slope of the straight line indicated the magnitude of the causal association.

Leave-one-out plot to visualize causal effect of interstitial lung disease or sarcoidosis on potential causal proteins when leaving one SNP out

**Fig.S3 The Forest plots of potential causal proteins inversely associated with ILD or its subtypes in bidirectional Mendelian randomization.**

1. **Forest plot of interstitial lung disease/idiopathic pulmonary fibrosis and BRSK2**


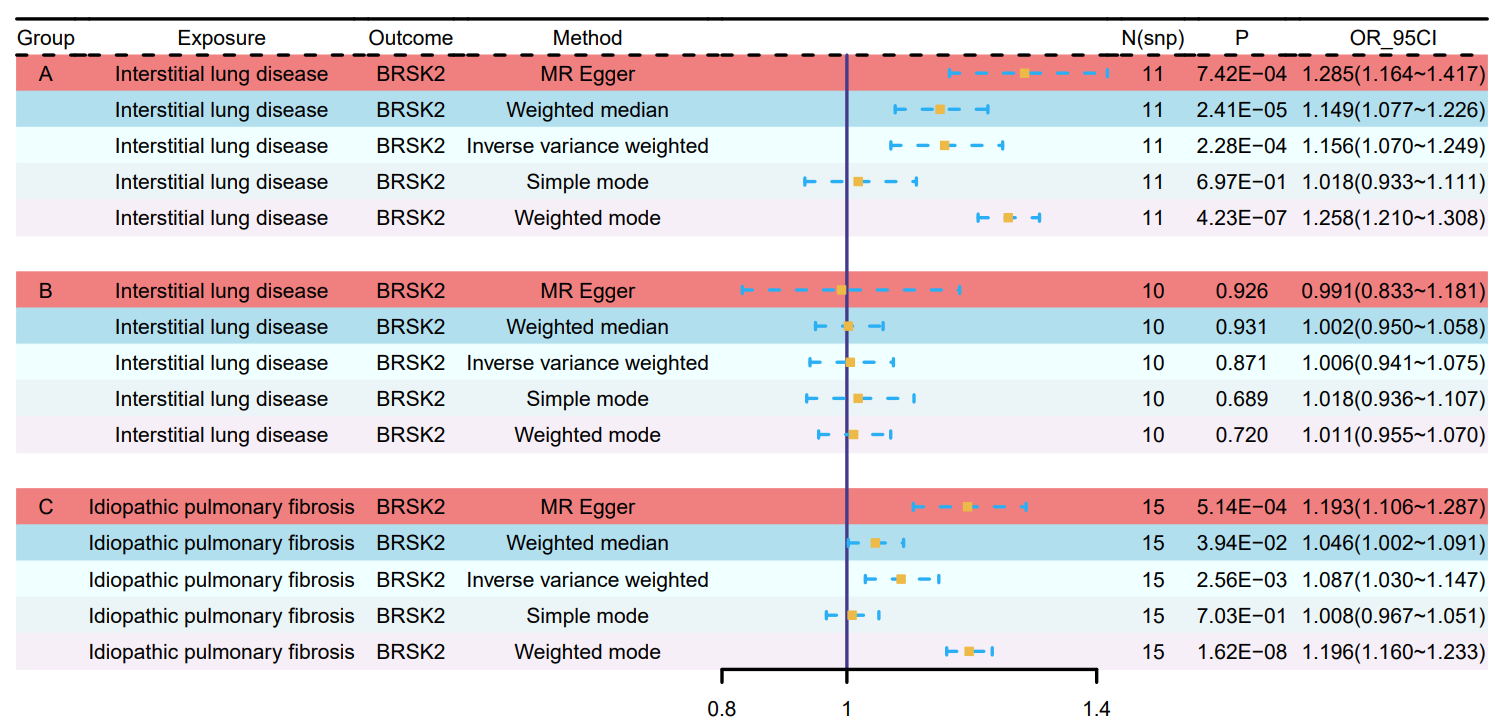


**(B) Forest plot of sarcoidosis and ANGPTL4**


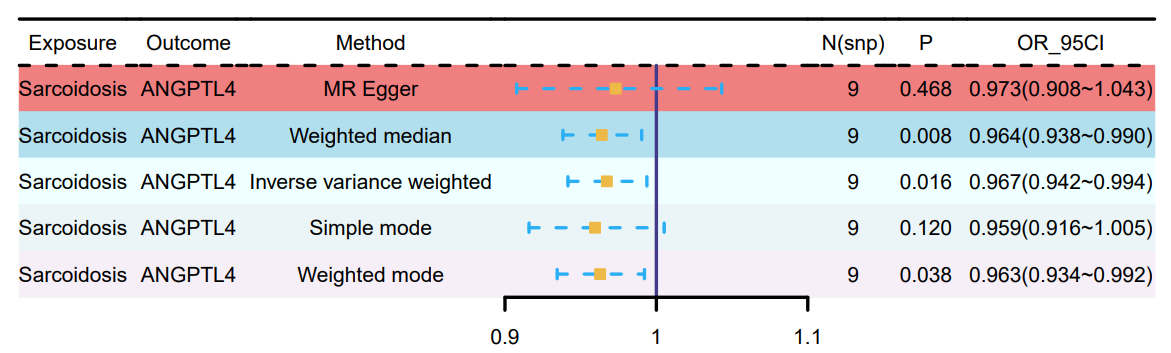


Forest plot to visualize causal effect of ILD or its subtypes on potential causal proteins. IVW indicated inverse-variance weighted, and random-effects model was used.

**Fig. S4 Bayesian colocalization analysis of potential causal proteins and Interstitial lung disease or its subtypes**.

1. **ADAM15-Idiopathic pulmonary fibrosis**


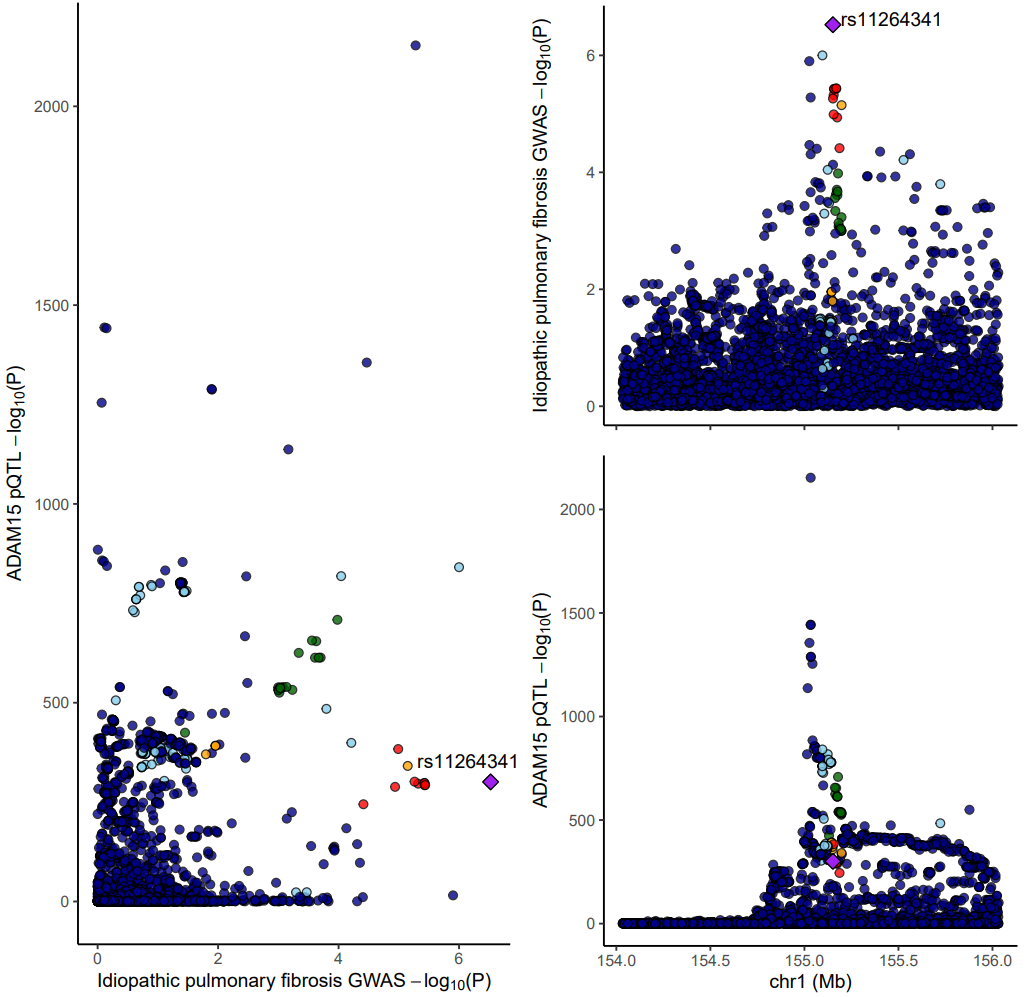


1. **ANGPTL4-Sarcoidosis**


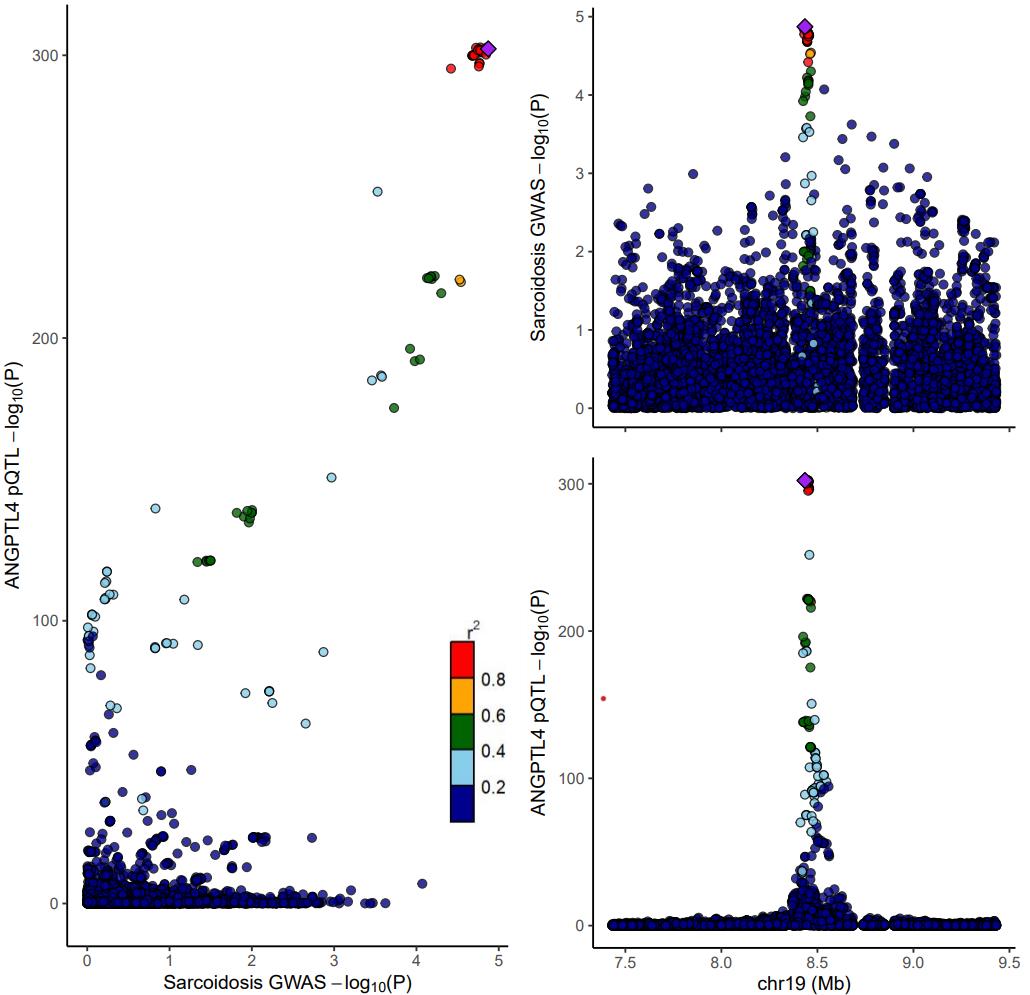


Colocalization analysis of ADAM15-Idiopathic pulmonary fibrosis (A) and ANGPTL4-Sarcoidosis(B). Diamond purple points represented the SNP that with the minimal sum of P value in corresponded protein GWAS and ILD or its subtypes GWAS.

**Fig.S5 The heterogeneity and pleiotropy analysis of priority proteins.**

1. **Scatter plot of ADAM15 and CDH15**


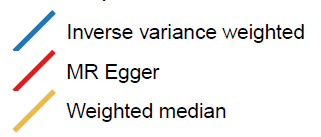

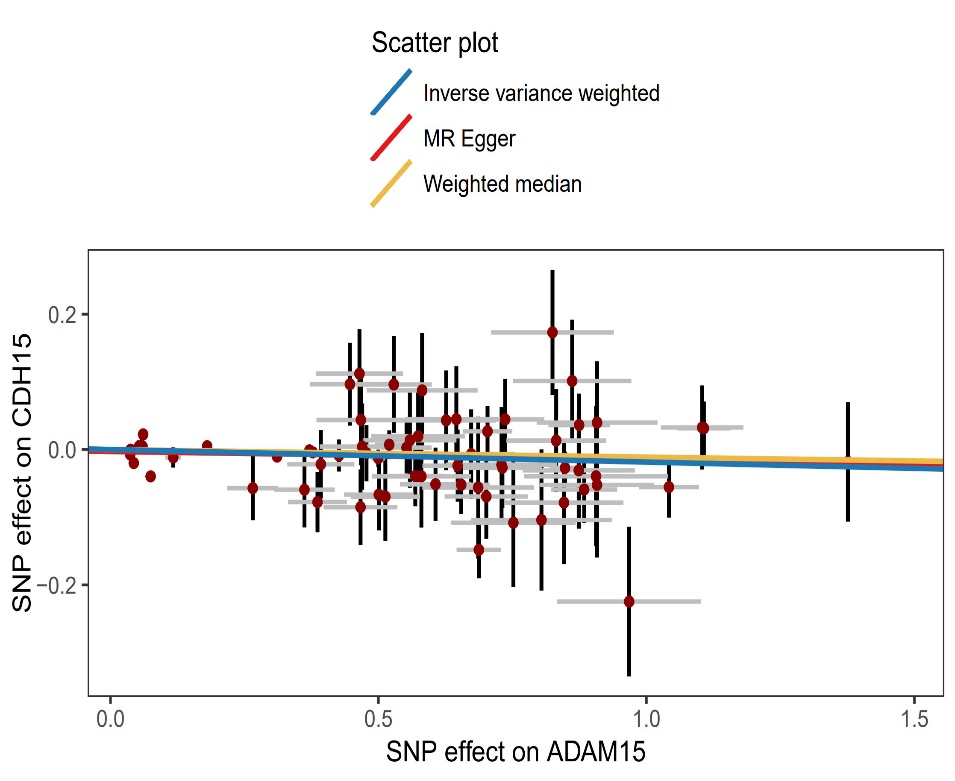


1. **Scatter plot of ADAM15 and CDH1**


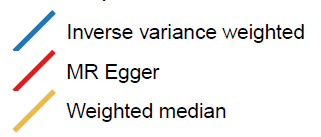

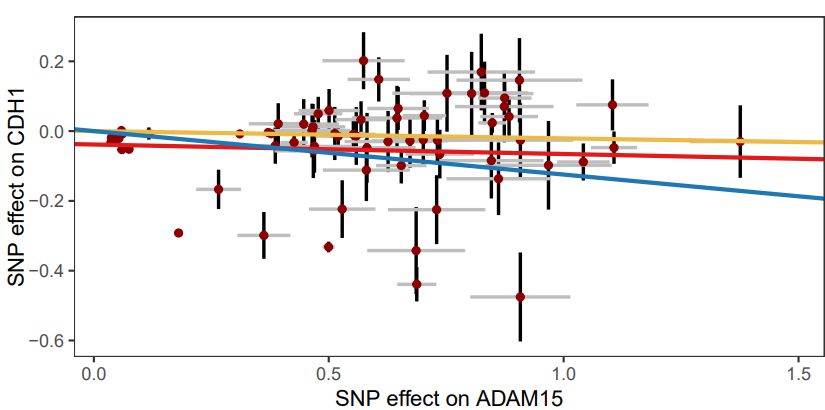


1. **Leave-one-out plot of ADAM15 and CDH1**

**
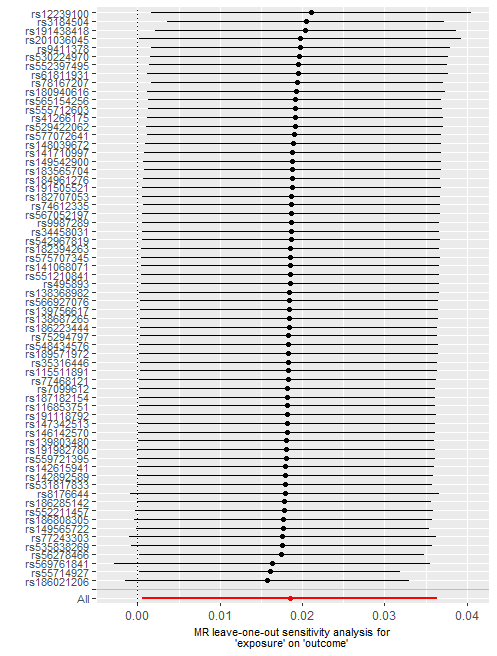
**

1. **Scatter plot of CDH1 and ADAM15**

**
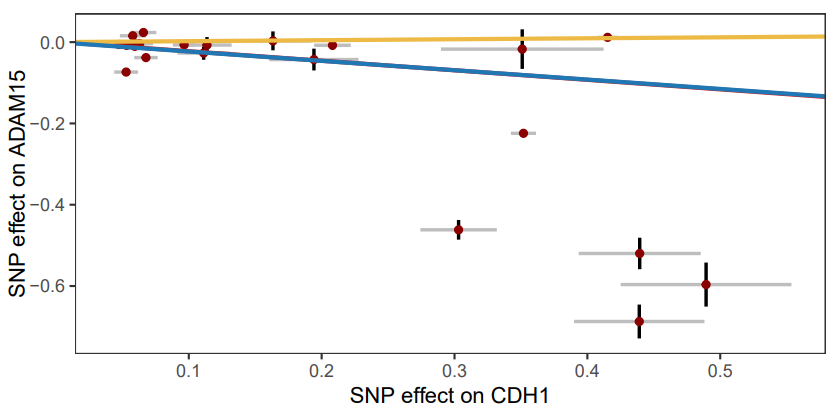
**
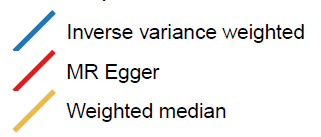


**(E) Leave-one-out plot of CDH1 and ADAM15**


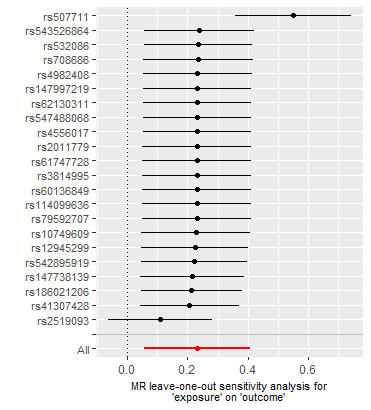


Scatter plot to visualize causal effect of priority proteins. The slope of the straight line indicated the magnitude of the causal association.

Leave-one-out plot to visualize causal effect of priority proteins when leaving one SNP out

**Fig. S6 The Forest plots of priority proteins.**

**(A) Forest plot of ADAM15 and CDH15**

**
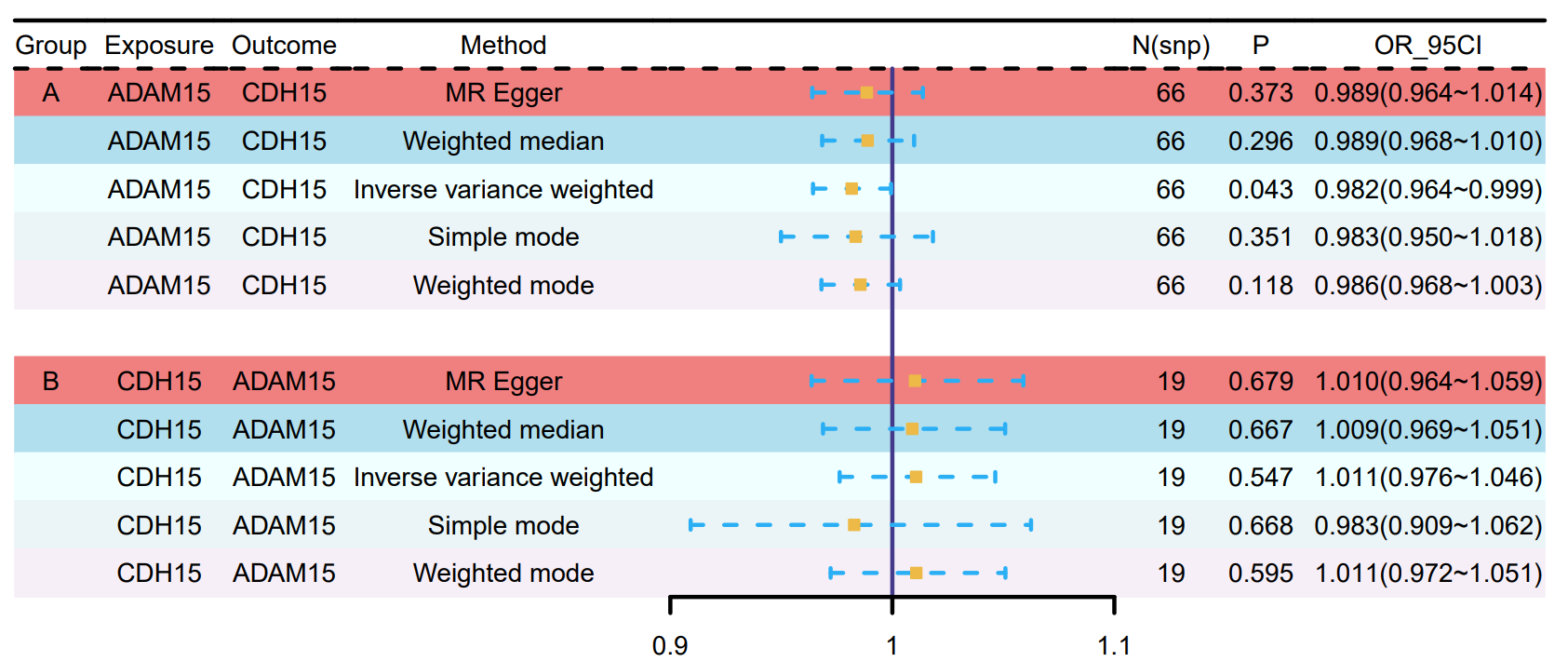
**

**(B) Forest plot of ADAM15 and CDH1**


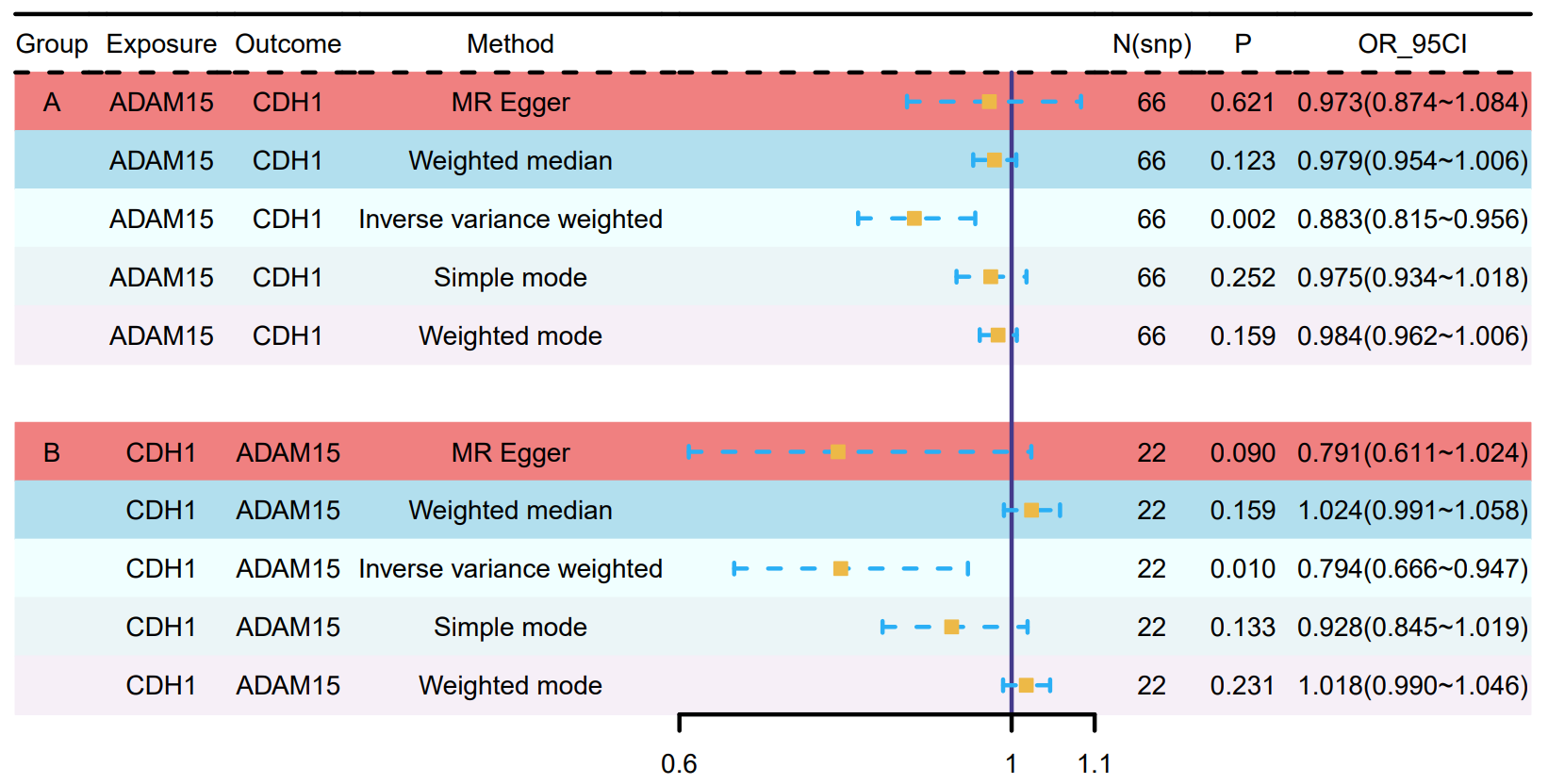


Forest plot to visualize causal effect of priority proteins on each other. IVW indicated inverse-variance weighted, and random-effects model was used.
